# Attachment Capability of Antagonistic Yeast *Rhodotorula glutinis* to *Botrytis cinerea* Contributes to Biocontrol Efficacy

**DOI:** 10.3389/fmicb.2016.00601

**Published:** 2016-05-03

**Authors:** Boqiang Li, Huaimin Peng, Shiping Tian

**Affiliations:** ^1^Key Laboratory of Plant Resources, Institute of Botany, Chinese Academy of SciencesBeijing, China; ^2^University of Chinese Academy of SciencesBeijing, China

**Keywords:** attachment, biological control, EMS mutagenization, gray mold, yeast

## Abstract

*Rhodotorula glutinis* as an antagonism show good biocontrol performance against various post-harvest diseases in fruits. In the present study, strong attachment capability of *R. glutinis* to spores and hyphae of *Botrytis cinerea* was observed. Further analysis showed that certain protein components on the yeast cell surface played critical role during the interaction between *R. glutinis* and *B. cinerea*. The components mainly distributed at the poles of yeast cells and might contain glycosylation modification, as tunicamycin treated yeast cells lost attachment capability to *B. cinerea*. To investigate contributions of attachment capability of *R. glutinis* to its biocontrol efficacy, yeast cells were mutagenized with 3% methane-sulfonic acid ethyl ester (EMS), and a mutant CE4 with stable non-attaching phenotype was obtained. No significant difference was found on colony, cell morphology, reproductive ability, and capsule formation between the mutant and wild-type. However, there was a distinct difference in India ink positive staining patterns between the two strains. Moreover, wild-type strain of *R. glutinis* showed better performance on inhibiting spore germination and mycelial growth of *B. cinerea* than CE4 strain when yeast cells and *B. cinerea* were co-cultured *in vitro*. In biocontrol assay, both wild-type and CE4 strains showed significant biocontrol efficacy against gray mold caused by *B. cinerea* in apple fruit, whereas, control effect of CE4 strain was lower than that of wild-type. Our findings provided new evidences that attachment capability of *R. glutinis* to *B. cinerea* contributed to its biocontrol efficacy.

## Introduction

Post-harvest decay of fruits and vegetables results in major economic losses. By far, the use of synthetic fungicide remains the main method for controlling post-harvest diseases (Droby et al., 2009). However, due to increasing public concern on environmental pollution and food safety causing by fungicide residues, some new strategies were developed as alternatives to manage post-harvest decay. Among them, biological control using microbial antagonists was considered as one of the most promising ways (Spadaro and Gullino, 2004; Liu et al., 2013).

In comparison with other microbial antagonists, yeasts have a number of attributes which make them suitable as biocontrol agents for post-harvest disease control, such as simple nutritional requirements, survival in adverse environmental conditions, good performances against a wide range of pathogens on different commodities, and compatibility with commercial processing procedures (Droby et al., 2009). Many yeast species have been reported to be effective biocontrol agents against post-harvest diseases (Wisniewski et al., 2007; Sharma et al., 2009; Liu et al., 2013). Action modes of antagonistic yeasts include competition for space, nutrition and iron, formation of biofilm, production of antifungal diffusible and volatile metabolites, induction of host resistance, and mycoparasitism (Spadaro and Droby, 2016). Among them, mycoparasitism is interesting, in which antagonistic yeasts show capability of attaching tenaciously to fungal pathogens. Allen et al. (2004) tested attachment capability of 294 phylloplane yeast isolates to spores of *Botrytis cinerea* and found that 260 isolates showed positive attachment. Among these isolates, several yeast species were reported as biocontrol agents, such as *Cryptococcus laurentii*, *Pichia guilliermondii*, and *Rhodotorula glutinis*, and showed good performance against various post-harvest pathogens (Yu et al., 2007, 2013; Zhang et al., 2010, 2011; Lahlali et al., 2014; Yan et al., 2014). With attachment, antagonistic yeasts could cause pitting and collapse on the surface of pathogen mycelium, and severe internal injury, such as protoplasm degeneration (Wisniewski et al., 1991; El-Ghaouth et al., 1998). Mycelial degradation may be associated with the secretion of chitinase, glucanase, and protease from antagonistic yeasts (Chan and Tian, 2005; Zhang et al., 2011; Banani et al., 2014). In addition, the attachment capability of antagonistic yeasts may play a key role by either enhancing nutrient competition or some other undetermined mechanism (Wisniewski et al., 1991). Although attachment of antagonistic yeasts to fungal pathogens is considered as contributing to the biocontrol efficacy, few data by far has indicated that how important the attachment capability is.

*Botrytis cinerea*, a necrotrophic fungal pathogen, causes gray mold disease in more than 200 host plant species and is especially destructive on fruits and vegetables (Williamson et al., 2007). The pathogen is capable of transferring sRNA effectors into host plant cells to suppress host immunity and secreting a large set of enzymes to kill host cells during the infection process (Li et al., 2012; Weiberg et al., 2013). Fungicide-resistant strains of *B. cinerea* frequently emerge in field, and alternatives are needed to effectively control the pathogen. It has been reported that essential oils obtained from aromatic plants could markedly inhibit spore germination and mycelial growth of *B. cinerea in vitro*, and showed good protection against gray mold disease in tomato under greenhouse condition (Soylu et al., 2010). Besides essential oils, biological control is also considered as a promising way for control of *B. cinerea*. As an antagonistic agent, *R. glutinis* has presented good biocontrol efficacy against gray mold in different fruits (Zhang et al., 2009; Ge et al., 2010) and other post-harvest diseases caused by *Penicillium expansum* (Chen et al., 2015), *Alternaria alternata* (Yan et al., 2014), *Rhizopus stolonifer* (Zhang et al., 2010). In our preliminary study, a strain of *R. glutinis* isolated in our previous study (Qin et al., 2003) was found to exhibit strong attachment capability to spores and hyphae of *B. cinerea*.

In order to investigate the role of attachment of the yeast strain in biocontrol efficacy, we generated a non-attaching mutant by EMS mutagenesis, and compared biological characteristics and biocontrol efficacy between non-attaching mutant and wild-type.

## Materials and Methods

### Yeast Strain

*Rhodotorula glutinis* (Fresen.) F.C. Harrison was isolated in the previous experiment and identified by CABI Bioscience (Egham, UK) (Qin et al., 2003). The strain was preserved in glycerol at -80°C. Before the experiment, it was inoculated and maintained in yeast peptone dextrose (YPD) medium at 26°C.

### Pathogen

*Botrytis cinerea* B05.10 was kindly provided by Dr. Tudzynski (Westfälische Wilhelms-Universität Münster, Germany). The pathogen was maintained on potato dextrose agar (PDA) at 4°C. For the production of *R. glutinis* non-attaching mutants, mycelia were collected after *B. cinerea* was cultured for 2 weeks at 22°C on PDA. For attachment and biocontrol assays, spores were collected and resuspended with sterile distilled water.

### Fruit

‘Fuji’ apples (*Malus domestica* Borkh.) with commercial maturity and without wounds or rot were classified according to their uniformity of size. Then, the selected fruit were surface-disinfected with 2% (v/v) sodium hypochlorite for 2 min, washed with tap water and air-dried prior to use (Yao et al., 2004).

### Attachment Assay between *R. glutinis* and *B. cinerea*

The yeast cells cultured in YPD medium were collected at late log-phase, resuspended to 2 × 10^8^ cells/mL after washing three times using sterile distilled water. Then, aliquot of yeast cell suspension was mixed with equal volume of *B. cinerea* spore suspension at the concentration of 1 × 10^7^ spores/mL. After vortex for 30 s, aliquot of 20 μL of the mixture was loaded onto a haemocytometer, and observed under Zeiss Axioskop 40 microscope (Carl Zeiss, Oberkochen, Germany). To facilitate the observation on the budding sites of yeast cells, fluorescent dye calcofluor white (Sigma, Saint Louis, MO, USA) was used at the concentration of 100 μg/mL.

### Scanning Electron Microscopy (SEM) Observation

The yeast cells cultured in YPD medium were collected at late log-phase, resuspended to 2 × 10^8^ cells/mL after washing three times using 0.1 M PBS (pH7.4). Then, aliquot of yeast cell suspension was mixed with equal volume of *B. cinerea* spore suspension at the concentration of 1 × 10^7^ spores/mL. After vortex for 30 s, the mixture was centrifuged (300 g, 15 s), the supernate containing un-attached yeast cells was removed. The pellet was gently resuspended and fixed with 100 μL glutaraldehyde solution (2.5%) for 2 h at 25°C. After centrifugation (300 g, 15 s) and washing twice with dd H_2_O, post-fixation was performed with 1% osmium acid for 1 h at 25°C. The fixed sample was washed three times with dd H_2_O, and completely dried in a vacuum centrifuge. Then, the sample was coated with gold–palladium and observed under Hitachi S-4800 SEM (Japan).

### Effects of Cell Surface Extract of *R. glutinis* on Attachment between Yeast Cells and Spores of *B. cinerea*

The yeast cells cultured in YPD medium for 48 h were collected, resuspended to 1 × 10^9^ cells/mL after washing three times using sterile distilled water. Aliquots of 1 mL yeast cell suspension were added into 1.5-mL EP tubes, and vibrated using cell disruptor (Disruptor Genie, Scientific Industries, New York, NY, USA) for 20 min (2 min each time with interval of 2 min in ice bath, a total of 10 times). After centrifugation (10,000 *g*, 5 min), the supernate was collected as cell surface extract (CSE). The spores of *B. cinerea* were suspended with CSE at concentration of 1 × 10^7^ spores/mL. After incubated for 30 min at 25°C, spores were mixed with equal volume of 2 × 10^8^ cells/mL yeast cell suspension. The attachment between yeast cells and spores of *B. cinerea* was examined under microscope. To determine the role of proteins in CSE on inhibiting attachment between yeast cells and spores of *B. cinerea*, CSE was digested with Pronase E (Sigma, Saint Louis, MO, USA) at final concentration of 2 mg/mL for 1 h prior to attachment assay.

### Tunicamycin Treatment and FITC-ConA Fluorescence Staining

The yeast cells were collected after cultured for 48 h in YPD medium with or without 5 μg/mL tunicamycin (Sigma, Saint Louis, MO, USA). The collected cells were washed with lectin buffer (10 mM HEPES [pH 7.5], 0.15 M NaCl, 0.1 mM CaCl_2_, 0.01 mM MnCl_2_), and incubated with FITC-Con A (100 μg/mL) (Sigma, Saint Louis, MO, USA) for 30 min at 25°C. After washing twice with lectin buffer, yeast cells were examined under a Zeiss Axioskop 40 microscope equipped with a UV-light source using a 485-nm excitation and 530-nm emission filter combination. The attachment assay of yeast cells treated with or without tunicamycin was performed as described above.

### Production of *R. glutinis* Non-attaching Mutants

*Rhodotorula glutinis* non-attaching mutants were obtained according to the method of Buck and Andrews (1999a) with some modifications. Briefly, yeast cells were collected at late log-phase, and mutagenized with 3% methane-sulfonic acid ethyl ester (EMS) for 40 min, which killed approximately 50% of the initial population. Following mutagenesis, the cells were divided into three fractions (Fraction A, B, and C). Each fraction representing a separate mutagenized population was cultured in YPD. After 24 h, each culture containing mutagenized cells was collected into centrifuge tube by centrifugation (3,000 *g*, 5 min), washed and resuspended with sterile distilled water. Then, mycelia of *B. cinerea* were added to each tube, and the mixture was vortexed for 1 min. With centrifugation (500 g, 30 s), pathogen mycelia with attached yeast cells were pelleted, and non-attaching yeast cells in the supernate were transferred. The procedure was repeated for at least 4 times to enrich non-attaching cells. The final, non-attaching fraction was plated onto YPD. From plates representing a separate initial population of mutagenized cells, colonies were selected and retested to confirm the loss of attachment capability as described above. Then, candidate isolates were purified by a single cell isolation technique and successively cultured for at least five times to evaluate the stability of non-attaching phenotype.

### Determination of Biological Characteristics of Wild-Type and Non-attaching Mutant

Wild-type and mutant of *R. glutinis* were cultured with YPD medium with or without 2% agar, biological characteristics of colony, cell morphology and growth curve were determined according to methods described elsewhere. In the assay of growth curve, the initial cell concentration of each yeast strains was 1 × 10^5^ cells/mL, the wavelength for determining OD value of cell cultures was 600 nm.

Surface staining patterns and EPS capsule of wild-type and mutant of *R. glutinis* were assayed with the India ink positive and negative staining methods reported by Buck and Andrews (1999a,b). For inducing formation of EPS capsule, yeast carbon base medium (YCB, Difco) with 3% glucose was used.

### Effects of Wild-Type and Non-attaching Mutant on Spore Germination of *B. cinerea In Vitro*

The yeast cells and spores of *B. cinerea* were collected as described above. Yeast cells and spores of *B. cinerea* were mixed and resuspended in sterile 10% apple juice at final concentrations of 5 × 10^7^ cells/mL and 2.5 × 10^6^ spores/mL, respectively. Aliquot of 100 μL water agar (2%) was added on the center of a sterile glass slide. After 5 min, 25 μL of the mixture of yeast cells and *B. cinerea* spores was spread on water agar. Suspension containing only *B. cinerea* spores was used as the control. Then, each glass slide was placed in a 9-cm petri dish with a moist filter paper at the bottom. Petri dishes were incubated at 22°C for 10 h after which germination rate of *B. cinerea* spores was examined. Each treatment contained four replications, and at least 200 spores were examined in each replication.

### Effects of Wild-Type and Non-attaching Mutant on Mycelial Growth of *B. cinerea In Vitro*

Yeast cells and spores of *B. cinerea* were mixed and resuspended with sterile distilled water at final concentrations of 5 × 10^7^ cells/mL and 2.5 × 10^6^ spores/mL, respectively. Agar disks (5 mm diameter) were cut and removed from PDA plates (5 cm diameter). After vortex for 30 s, aliquot of 25 μL of the mixture was added into each well on the PDA plates. PDA plates inoculated 25 μL spore suspension of *B. cinerea* were used as the control. The plates were cultured at 22°C for 60 h after which colony diameter (extension of colony minus the PDA well diameter of 5 mm) was measured. Each treatment contained four replications.

### Biocontrol Assays

Four wounds (uniform 4 mm deep × 3 mm wide) were made on the cheek of each apple fruit with a sterile nail. Wild-type and CE4 strain of *R. glutinis* were collected after culturing for 24 h, resuspended to 1 × 10^8^ cells/mL after washing three times using sterile distilled water. The suspension of yeast cells was mixed with equal volume of *B. cinerea* spore suspension of 2 × 10^5^ spores/mL. Then, aliquots of 10 μL mixture were inoculated to the wound of apple fruit, respectively. Fruit inoculated with 10 μL spore suspension of *B. cinerea* at 1 × 10^5^ spores/mL were used as the control. Treated fruit were put in 400 × 300 × 100 mm plastic box, which was covered with a high density polyethylene bag to keep high humidity and stored at 22°C. Disease incidence and lesion diameter were determined each day until 5 days. Each treatment contained three replicates of eight fruits.

### Data Analysis

Statistical analyses were performed with SPSS version 11.5 (SPSS Inc., Chicago, IL, USA) and analyzed by one-way analysis of variance (ANOVA). Mean separations were performed by Duncan’s multiple range tests. Differences at *P* ≤ 0.05 were considered significant.

## Results

### Attachment between *R. glutinis* and *B. cinerea*

**FIGURE 1:**
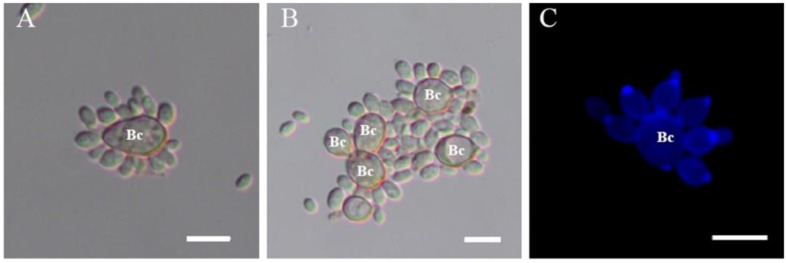
**Attachment assay of *Rhodotorula glutinis* to spores of *Botrytis cinerea*.** **(A,B)** Attachment between *R. glutinis* and spores of *B. cinerea* was observed under light field; **(C)** Yeast cells mostly attached to spores by the poles of cells, nearby the budding sites which were stained by fluorescent dye calcofluor white. Bc indicates spores of *B. cinerea*. Bar = 10 μm.

**FIGURE 2:**
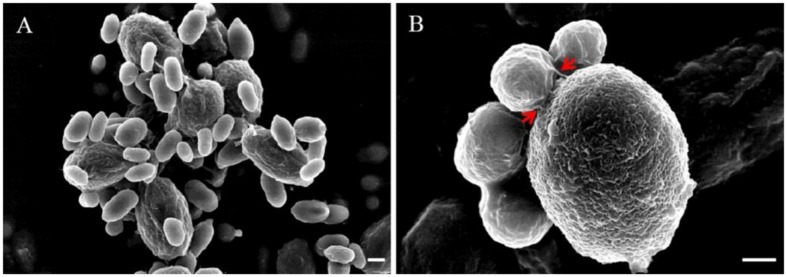
**Attachment of *R. glutinis* and spores of *B. cinerea* using scanning electron microscopy (SEM).** **(A)**, Attachment between *R. glutinis* and spores of *B. cinerea* was observed under SEM; **(B)**, Close-up view showing the attachment between *R. glutinis* and *B. cinerea*. Red arrows indicate fibers linking yeast cell and spore of *B. cinerea*. Bar = 1 μm.

When yeast cells of *R. glutinis* and spores of *B. cinerea* were mixed together, transient and strong attachment between them was observed (**Figure 1**). Spores of *B. cinerea* were tightly surrounded by yeast cells (**Figure 1A**), and a number of spores and yeast cells aggregated together sometimes (**Figure 1B**). Interestingly, yeast cells mostly attached to spores by the poles of cells, nearby the budding sites (**Figure 1C**). SEM observation suggested that fibers on the cell surface might play important role in the attachment between yeast cells and spores of *B. cinerea* (**Figure 2**). The attachment was also observed between yeast cells of *R. glutinis* and mycelia of *B. cinerea* or other fungal pathogens, such as *Monilinia fructicola* (data not shown).

### Effects of CSE on Attachment between *R. glutinis* and *B. cinerea*

**FIGURE 3:**
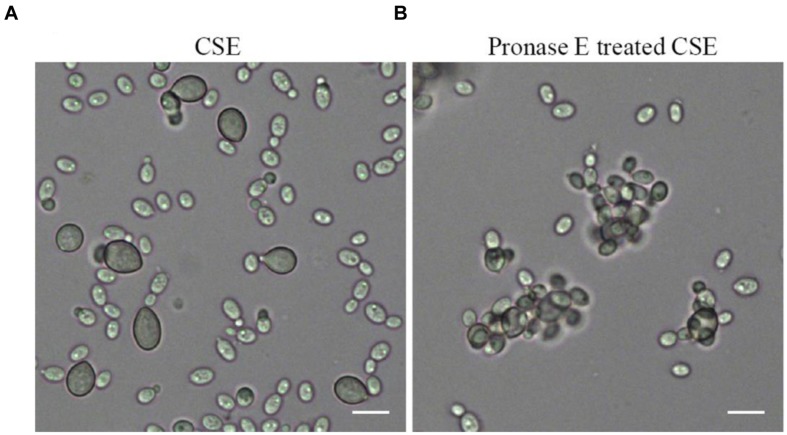
**Effects of cell surface extract (CSE) of *R. glutinis* on attachment between yeast cells and spores of *B. cinerea*.** Firstly, spores of *B. cinerea* were incubated with CSE **(A)** or Pronase E treated CSE **(B)**, then attachment between yeast cells and spores of *B. cinerea* was examined. Bar = 10 μm.

The attachment capability of *R. glutinis* was vibration-sensitive. The yeast nearly completely lost attachment capability to spores of *B. cinerea* when yeast cells were pre-treated with vibration for 20 min (data not shown). The result suggested that certain components on the cell surface played critical role during the interaction between *R. glutinis* and *B. cinerea* and exfoliated from cells during vibration. Incubating spores of *B. cinerea* with CSE markedly inhibited the attachment between *R. glutinis* and *B. cinerea* (**Figure 3A**). While, the influence of CSE was impaired after it was digested using Pronase E (**Figure 3B**).

### Effects of Tunicamycin on Attachment between *R. glutinis* and *B. cinerea*

**FIGURE 4:**
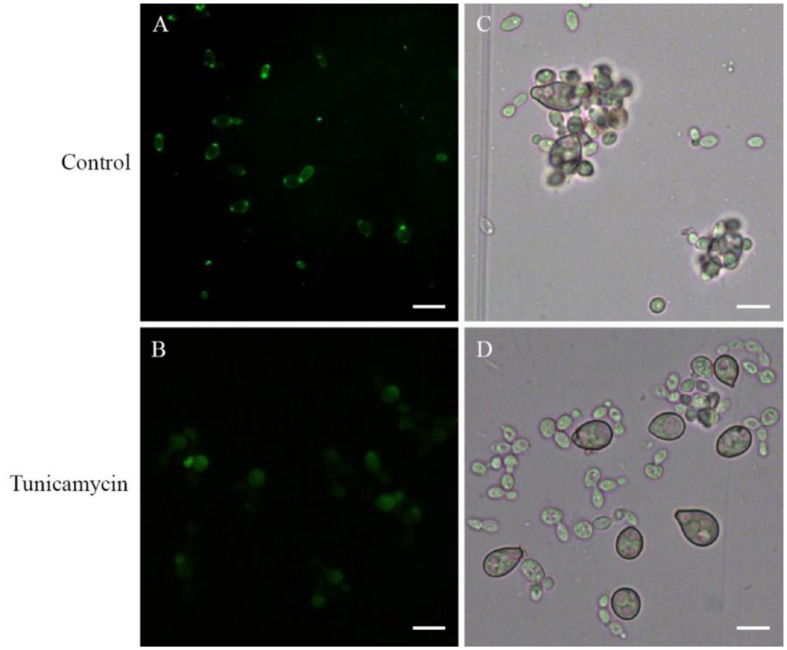
**Effects of tunicamycin on attachment between *R. glutinis* and *B. cinerea*.** **(A,B)** FITC-Con A staining of yeast cells treated or un-treated with 5 μg/mL tunicamycin. **(C,D)** Attachment assay of yeast cells treated or un-treated with tunicamycin. Bar = 10 μm.

Con A as a lectin can bind specifically to α-Mannose or α-Galactose structures in glycoproteins. So the FITC-ConA fluorescence staining could be used to detect the distribution of glycoproteins on cell surface of *R. glutinis*. FITC-Con A fluorescence occurred over the entire cell surface and was very intense at the poles of cells (**Figure 4A**). The strong polar FITC-Con A staining pattern was not observed when *R. glutinis* grew in YPD containing 5 μg/mL tunicamycin (**Figure 4B**). Further, the tunicamycin treated yeast cells lost attachment capability to spores of *B. cinerea* (**Figures 4C,D**). It was also found that tunicamycin treated yeast cells reproduced slowly and presented abnormal cell shape.

### Isolation and Characterization of Non-attaching Mutants

**FIGURE 5:**
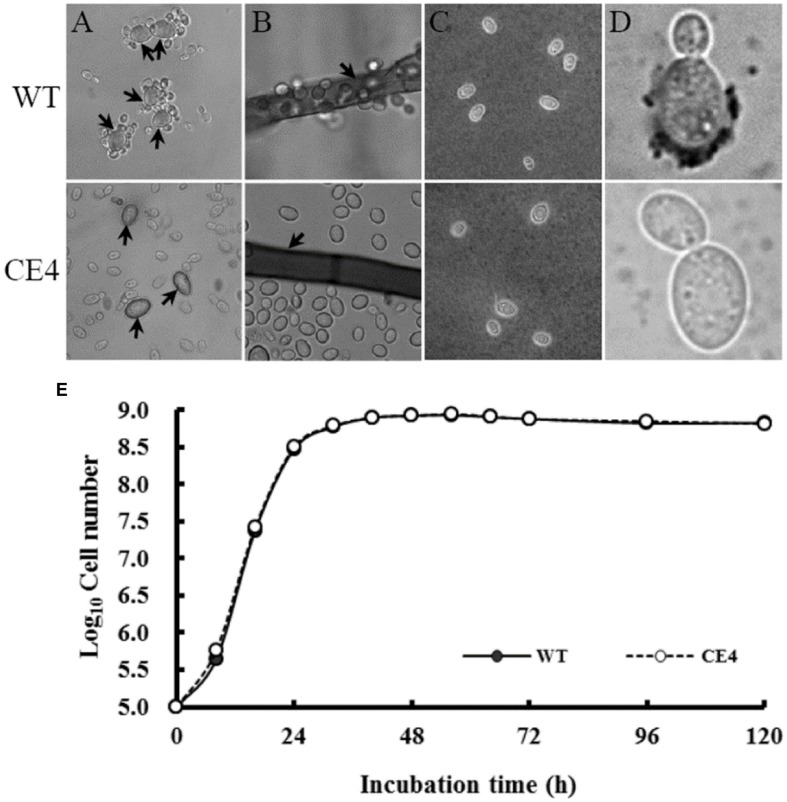
**Attachment assay and determination of biological characteristics of *R. glutinis* wild-type (WT) and non-attaching mutant (CE4).** **(A)** attachment assay between *R. glutinis* and spores of *B. cinerea* (arrows indicating spores of *B. cinerea*); **(B)** Attachment assay between *R. glutinis* and hyphae of *B. cinerea* (arrows indicating hyphae of *B. cinerea*); **(C)** India ink staining positive patterns; **(D)** India ink staining negative patterns; **(E)** Growth curves of WT and CE4 strains.

By mutagenizing with EMS, seven non-attaching mutants were obtained from independent initial mutagenized populations. Among them, three mutants were isolated from Fraction A, one mutant from Fraction B and two mutants from Fraction C. However, there was only one mutant (CE4, from Fraction C) showed stable non-attaching phenotype to *B. cinerea* after purification and successive culture (**Figures 5A,B**). Subsequently, we compared the difference in colony, cell morphology, and reproductive ability between CE4 and wild-type strains cultured in YPD medium. No significant difference in colony appearance and color, cell shape and size were observed. Both CE4 and wild-type strains possessed the capability of EPS capsule formation when cultured in YCB medium and negatively stained with India ink (**Figure 5C**). However, in the assay of India ink positive staining, a distinct difference between the two strains was found, i.e., India ink could stain the poles of wild-type cells, rather than CE4 cells (**Figure 5D**). In addition, growth curve of the two strains nearly completely overlapped, indicating the two strains has similar reproductive ability (**Figure 5E**).

### Effects of Wild-Type and Non-attaching Mutant on Spore Germination and Mycelial Growth of *B. cinerea*

**FIGURE 6:**
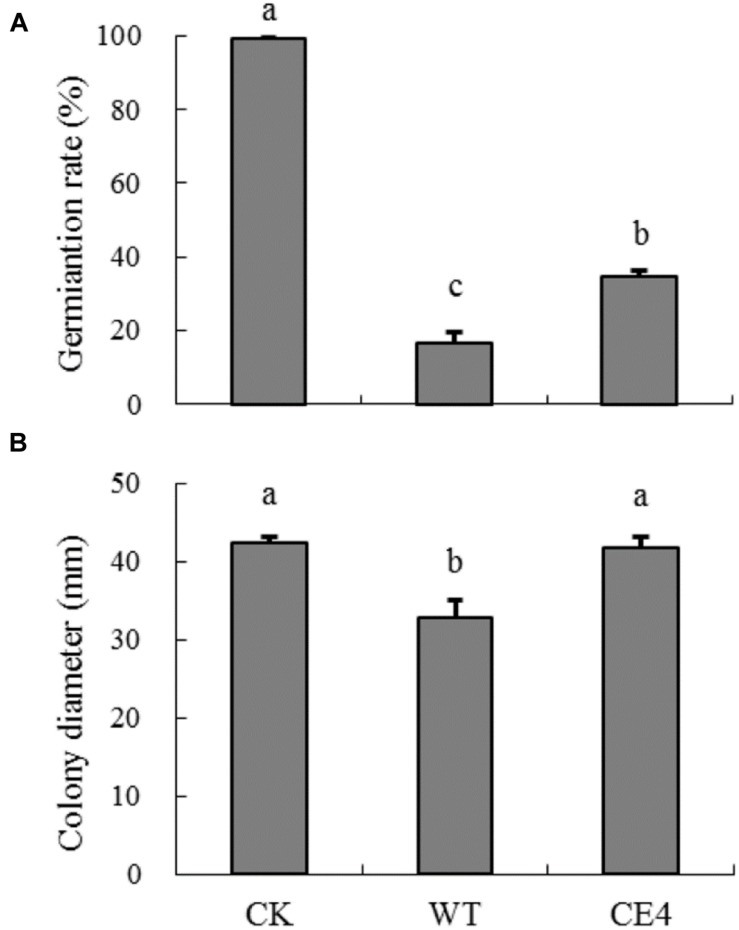
**Effects of wild-type and non-attaching mutant on spore germination and mycelia growth of *B. cinerea*.** **(A)** Germination rate after 10 h at 22°C; **(B)** Colony diameter after 60 h at 22°C. Error bars indicate standard deviations. Values followed by different letters are significantly different according to Duncan’s multiple range test (*P* < 0.05).

After 10 h, germination rate of *B. cinerea* spores reached to nearly 100% in control treatment (**Figure 6A**). Co-culture of yeast cells and *B. cinerea* spores significantly inhibited spore germination. The germination rate of *B. cinerea* spores in WT treatment was about half of that in CE4 treatment (17 vs. 35%). Co-culture of wild-type yeast cells and *B. cinerea* also inhibited colony extension (**Figure 6B**). In contrast, CE4 strain had no significant effect on mycelial growth of *B. cinerea*.

### Biocontrol Efficacy of Wild-Type and Non-attaching Mutant

**FIGURE 7:**
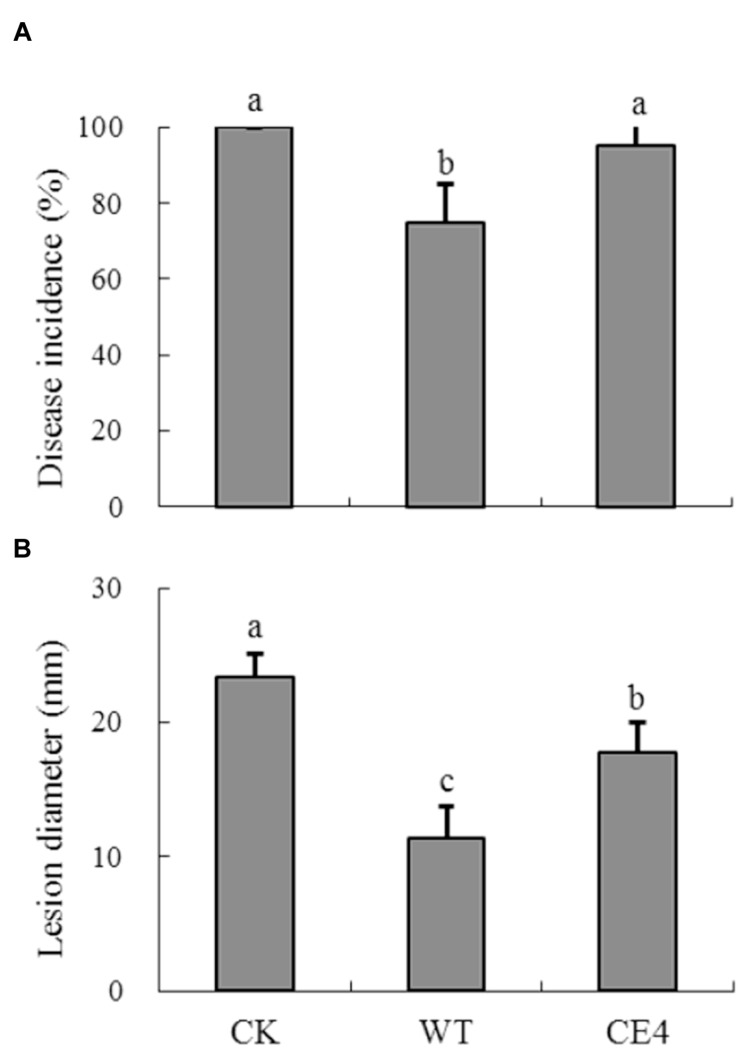
**Biocontrol efficacy of *R. glutinis* wild-type (WT) and non-attaching mutant CE4 against *B. cinerea* in apple fruit.** **(A)** Disease incidence; **(B)** Lesion diameter. Error bars indicate standard deviations. Values followed by different letters are significantly different according to Duncan’s multiple range test (*P* < 0.05).

After stored 5 days at 22°C, disease incidence of gray mold cause by *B. cinerea* reached 100% in control fruit, and the average lesion diameter was 23.4 mm (**Figure 7**). Inoculation of both wild-type and CE4 strains reduced the disease incidence and lesion diameter in apple fruit. However, fruit treated with wild-type strain showed significantly lower disease incidence (75% vs. 95%) and smaller lesion diameter (11.4 mm vs. 17.8 mm) as compared with CE4 treatment (*P* < 0.05), indicating wild-type provided better control effect than CE4 strain.

## Discussion

*Rhodotorula glutinis* is a common inhabitant of phylloplane communities (Di Menna, 1959). The yeast has been reported to possess positive attachment capability to post-harvest fungal pathogens, which was considered as one of main biocontrol mechanisms of the biocontrol agent (Castoria et al., 1997; Allen et al., 2004). However, various modes of action may simultaneously involve in the interaction between antagonisms and pathogens. Thus, it is difficult to evaluate the contribution of each action mode possessed by the antagonistic yeast. Isolating non-attachment mutants should be a good method to study the importance of attachment.

*Rhodotorula glutinis* is haploid and suitable for mutagenesis (Tully, 1985). In the present study, we mutagenized *R. glutinis* wild-type strain by EMS. The method was also used by Buck and Andrews (1999a) on a strain of basidiomycetous yeast *Rhodosporidium toruloides* (anamorph, *R. glutinis*). During the processes of screening non-attaching mutants, we used mycelia of *B. cinerea* to exclude attached yeast cells instead of polystyrene petri dishes. Because cells of wild-type can tenaciously attach to *B. cinerea* mycelia, the modification let us obtain non-attaching mutants more efficiently. The method may provide help for isolating non-attaching mutants in other yeast species. With the method, we obtained a mutant, CE4, which completely lost the attachment capability (**Figures 5A,B**). In addition, no significant differences in colony color, cell shape, and cell size were observed between CE4 and wild-type. Moreover, the two strains showed equal reproductive ability (**Figure 5E**). Reproductive ability of biocontrol agents is closely related with successful colonization in fruit wounds and important for biocontrol (Janisiewicz and Korsten, 2002). Interestingly, wild-type strain of *R. glutinis* showed better performance on inhibiting spore germination and mycelial growth of *B. cinerea* than CE4 strain when yeast cells were co-cultured with *B. cinerea in vitro* (**Figure 6**). Further, biocontrol efficacy of CE4 strain was significantly lower than that of wild-type strain, though CE4 strain also reduced the disease incidence and lesion diameter against gray mold caused by *B. cinerea* in apple fruit (**Figure 7**). These results suggested that the attachment of *R. glutinis* to *B. cinerea* contributed to the full biocontrol efficacy, and other modes of action were also involved. Competition for nutrition and space might be still the main mode of action for *R. glutinis* to control post-harvest diseases. In the biocontrol assay, wild-type strain showed about 25% efficacy of disease incidence after 5 days by comparing with control, which was relatively lower than that reported in previous studies (Zhang et al., 2009; Yan et al., 2014). The results might be caused by the lower ratio of yeast cells relative to *B. cinerea* spores (5 × 10^7^ cells/mL vs. 1 × 10^5^ spores/mL) in the present study. In addition, simultaneous inoculation of yeast cells and *B. cinerea* spores might also lead higher disease incidence than inoculation of yeast cells ahead of pathogen.

Important role of extracellular polymeric substances (EPS) in cell attachment has been well documented in *Candida* species (Seneviratne et al., 2008). For antagonistic yeasts, Andrews et al. (1994) reported that formation of EPS capsule contributed to attachment of *Aureobasidium pullulans*. Whereas, our results showed that both wild-type and non-attaching mutant CE4 could produce capsules by analysis of India ink negative staining (**Figure 5C**), indicating that produce of EPS capsule is not the prerequisite to cell attachment for yeasts. Similar result was also reported by Buck and Andrews (1999a).

Interestingly, we observed that yeast cells mostly attached to spores of *B. cinerea* by the poles of cells (**Figure 1**). At the same time, both FITC-Con A and India ink positive staining showed a strong polar pattern in the wild-type strain of *R. glutinis* (**Figures 4A** and **5D**), suggesting that certain components on cell poles might play an important role in the attachment. As attachment capability of *R. glutinis* was vibration-sensitive, the critical components might loosely bind to the cell surface. The components could be included in CSE by vibration treatment, and blocked the attachment sites on the surface of *B. cinerea* spores during incubation, finally inhibited the attachment between *R. glutinis* and *B. cinerea* (**Figure 3A**). On the contrary, CSE treated by Pronase E could not inhibit the attachment (**Figure 3B**), indicating some proteins were involved in the attachment. These proteins might be glycosylated, because tunicamycin, an inhibitor of *N*-glycoprotein synthesis, impaired the attachment capability of *R. glutinis* (**Figure 4D**). Critical roles of cell surface proteins, especially glycoproteins in adhesion interaction have been reported in previous studies (Teixeira et al., 2014; de Oliveira et al., 2015).

## Conclusion

A non-attaching mutant of the biocontrol yeast strain, *R. glutinis* CE4, was generated by a modified method of EMS mutagenesis. This mutant exhibited weaker ability on inhibiting spore germination and mycelial growth of *B. cinerea in vitro*, as well as lower biocontrol effect against *B. cinerea* in apple fruit, compared to the wild-type strain. These results suggested that attachment capability of *R. glutinis* to *B. cinerea* contributed to its biocontrol efficacy. Furthermore, certain protein components, which loosely bound to the cell surface and mainly located on cell poles, might play a critical role in the attachment between *R. glutinis* and *B. cinerea*. The components and involved molecular mechanisms are worth investigating in future study, which will provide help to enhance efficacy of biocontrol agents.

## Author Contributions

ST conceived and designed the experiments. BL and HP performed the experiments. BL analyzed the data. BL and ST drafted the manuscript. All authors read and approved the final manuscript.

## Conflict of Interest Statement

The authors declare that the research was conducted in the absence of any commercial or financial relationships that could be construed as a potential conflict of interest.
